# 

**Published:** 1788

**Authors:** 


### LONDON

### MEDICAL JOURNAL,

FOR THE YEAR 1788,

PART THE THIRD.

### CATALOGUE OF BOOKS.

1. THE Connexion of Life with Refpiration; or, An Experimental Inquiry into the Effects of Submerfion, Strangulation, and feveral Kinds of noxious Airs on living Animals: With an Account of the Nature of the Difeafe they produce; its Diftinction from Death itfelf; and the moft effectual Means of Cure. By *Edmund Goodwyn*, M. D. 8vo. *Johnfon*, London, 1788.

2. The Duties of a Regimental Surgeon confidered: with Obfervations on his general Qualifications, and Hints relative to a more refpectable Practice, and better Regulation of that Department. Wherein are interfperfed many medical Anecdotes, and Subjects difcuffed, equally interefting to every Practitioner. By *R. Hamilton*, M. D. of the Royal College of Phyficians, London; Member of the Medical and Phyfical Societies of Edinburgh, and of the Medical Society of London. 2 Vols. 8vo. *Johnfon*, London, 1788.

3. Thirty-eight Plates, with Explanations; intended to illuftrate Linnæus's Syftem of Vegetables, and particularly adapted to the Letters* on the Elements of Botany. By *Thomas Martyn*, B. D. F. R. S. Profeſſor of Botany in the Univerſity of Cambridge 8vo. *White*, London, 1788.

4. Elements of Natural Hiſtory and Chemiſtry: being the ſecond Edition of the Elementary Lectures on thoſe Sciences, firſt publiſhed in 1782, and now greatly enlarged and improved by the Author, M. *de Fourcroy*, Doctor of the Faculty of Medicine at Paris, of the Royal Academy of Sciences, &c. Tranſlated into Engliſh. With occaſional Notes and an hiſtorical Preface by the Tranſlator. 4 Vols. 8vo. *Robinſons*, London, 1788.

5. Experiments and Obſervations to inveſtigate, by chemical Analyſis, the medical Properties of the Mineral Waters of Spa and Aix-la-Chapelle in Germany; and of the Waters and Boue near St. Amand in French Flanders. By *John Aſh*, M. D. Fellow of the Royal College of Phyſicians, of the Royal Society, and of the Society of Antiquaries. 8vo. *Robſon*, London, 1788.

6. The Gentleman's Stable Directory; or, Modern Syſtem of Farriery. Comprehending the preſent entire improved Mode of Practice;

* See Vol. VI. page 332.

concontaining all the moſt valuable Preſcriptions and approved Remedies. By *William Taplin*, Surgeon. 8vo. *Kearſley*, London, 1788.

7. The Gentleman's Experienced Farrier; containing the Methods of Diet, Exerciſe, Bleeding, Purging, &c., of Horſes; the anatomical Parts deſcribed; the Diſorders incident to Horſes, and their reſpective Cures, &c. By *William Foſter*, Farrier. 8vo. *Robinſons*, London, 1788.

8. Diſſertatio Inauguralis, complectens de Typho quædam. Auctore *Gulielmo May*, Anglo. 4to. Lugd. Batav. 1787.

9. Tabulæ Anatomicæ ex archetypis egregii pictoris *Petri Berrettini* Cortonenſis expreſſæ, & in aes inciſæ. Opus chirurgis, & pictoribus apprime neceſſarium. Alteram hanc editionem recenſuit, nothas iconas expunxit, perpetuas explicationes adjecit *Franciſcus Petraglia*, Philoſophiæ & Medicinæ Profeſſor. Romæ, 1788. Fol. max.

10. Bibliotheca Medicinæ practicæ, qua Scripta ad Partem Medicinæ practicam facientia a Rerum Initiis recenſentur, Auctore *Alberto von Haller*, Domino quondam in Goumoens le Jux & Feudi in Eclagnens; Equite Stellæ Polaris; Præſide Societatis Regiæ Goettingenſis; Præſide

Præside Societatis œconomicæ Bernensis; Sodali Academiæ Regiæ Scientiarum Parisinæ; Academiæ Imp. Nat. Cur. Acad. Imp. Ruff. Boruff. Suec. Bonon. Arcadicæ; Academiæ Regiæ Chirurgorum Paris. Societ. Reg. Brit. Bot. Flor. Bavaricæ; Helveticæ; Coll. Med. Edinburgensium; in Supremo Senatu Reip. Bernensis Ducentumviro; ex ejus Schedis restituit, auxit & edidit *Joachim Diterich Brandis*, M. D. Medicus Hildesiensis. Tomus IV. ab Anno 1686 ad A. 1707. 4to. Basileæ, 1788.

This fourth volume contains all that can be collected from the manuscripts of the illustrious Haller towards the continuation of this truly valuable work; but the learned Editor, Dr. Brandis, who appears to be ably qualified for the task, has undertaken (as we learn from his preface) to bring it down to the present time, and hopes in about three years to be able to complete his engagement on this subject with the Public. In the course of this volume many articles are inserted which had escaped the notice of Haller. It closes with an Index of the names of the Authors that occur in this and the preceding volumes; and the Editor promises soon to add a more ample Index of the Contents of the Work.

11. Essai analytique sur l'Air pur, & les differentes especes d'Air. Par M. *de la Metherie*, Docteur en Medecine & Membre des Academies de Dijon & de Mayence. Seconde Edition. 8vo. 2 Tomes. Paris, 1788.

12. Krankheitsgeschichte des Hoechstseeligen Koenigs von Preussen Friedrich's des Zweyten Majestaet. Von *Christian Gottlieb Selle*. 12mo. Berlin, 1786.

This is a concise and well-written narrative of the last illness of the late King of Prussia by the physician who attended him. The disease was a dropsy of the chest and belly, which terminated fatally in about eleven months. The King having expressed a wish that his body might not be embalmed, or even opened after death, nothing more was permitted than to puncture the abdomen with a trocar, by which means about four quarts of water were drawn off.

13. Vermischte Beobachtungen aus der practischen Arzneykunde, Wundarzneykunst und Geburtshulfe; *i. e.* Miscellaneous Observations relative to the Practice of Physic, Surgery, and Midwifery. By *L. Zorn*, M.D. 8vo. Wurtzburg, 1787.

14. Versuch einer Medizinischen Ortbeschreibung der Stadt Regensburg, nebst einer kurzen Ueber-

Ueberficht der Krankheiten welche in den Jahren 1784, 1785, und 1786, daselbst geherrschet haben; *i. e.* An Attempt towards a medical Topography of the City of Ratisbon; together with a concise View of the Diseases that prevailed there in the years 1784, 1785, and 1786. By *Jacob Christian Gottlieb Schaeffers*, Physician at Ratisbon, Body Physician and Counsellor of State to His Serene Highness the Prince of Tour and Taxis. 8vo. Ratisbon, 1787.

15. *Torberni Bergman* Meditationes de Systemate Fossilium naturali, in usum Oryctologiæ Studiosorum iterum typis mandatæ. 8vo. Oxoniæ, 1788.

16. Prime linee di pratica medica, opera di *Guglielmo Cullen*, Professore di Medicina pratica nell' Universita d'Edimburgo, primo medico di sua Maesta per la Scozia, &c. tradotta dall' Inglese da *Federico Rossi*, Professore di Chirurgia, ed articchita d'annotazioni ad uso degli Studenti di Medicina nella regia Universita di Siena. Vol. I. 8vo. Siena, 1788.

17. Dell' Arte Ostetricia: fogli periodici con rami colorati; trimestre primo. 8vo. Bologna, 1788.

